# Association between bruxism and the use of aligners in orthodontics

**DOI:** 10.4317/jced.61951

**Published:** 2024-10-01

**Authors:** Tatyana Heleiwa-Ferioli, Susana de la Cruz Vigo

**Affiliations:** 1 Clinica Universitaria Odontológica de la Universidad Europea de Madrid

## Abstract

**Background:**

The objective of this study is to assess the relationship between bruxism and the use of Invisalign® transparent aligners, and to determine if they increase or decrease the action of bruxing with its signs and symptoms.

**Material and Methods:**

A sample of 100 adult patients undergoing orthodontic treatment with transparent Invisalign® aligners was studied, analyzing signs and symptoms of bruxism by personal survey together with a clinical examination on 3 occasions: Pre-treatment, at 3 months of treatment and at 6 months of treatment.

**Results:**

There is a statistically significant difference between the amount of bruxism and the use of Invisalign® type aligners, for all the variables analyzed except in the analysis of the increase in headaches.

**Conclusions:**

The frequency of bruxism does not depend on sex, but is related to age groups, the most affected being 28-36 years old patients. Statistically significant differences have been observed (*P*<.05) with notable reductions in: 1) Clenching and/or grinding sensation, 2) Sensation of contracted masticatory muscles, 3) Muscular pain of the masticatory muscles and 3) Pain of the temporomandibular joint. Additionally, 4) facial aesthetics and lip position experienced statistically significant differences (*P*<.05) without an increase or reduction being predominant. There is no relationship between the increase in headaches and the use of Invisalign® transparent aligners, as no statistically significant differences were found (*P*<.05).

** Key words:**Bruxism, survey, aligners, Invisalign, Masticatory muscles, Temporomandibular joint.

## Introduction

The use of conventional orthodontic appliances complicates dental hygiene, interfering with aesthetics and causing discomfort to the patient. Proffit *et al*. proposed that the ideal orthodontic appliance should be capable of providing light forces while having the potential to resist masticatory forces, be firmly retained, apply controlled force to each movement between visits, all while allowing for anchorage control (1). In 1945, Kesling (2) was the first to introduce the use of multiple aligners for the correction of crowding. Subsequently, Ponitz (3) announced the use of a removable plastic retainer (Essix®; Dentsply, York, PA, USA). A few years later, in the 1990s, Sheridan *et al*. (4) popularized the retainers in coordination with interproximal dental reduction (IPR). In 1997, a computer science specialist along with two students from Stanford University, Zia Chishti and Kelsey Wirth, founded the company Align Technology in Palo Alto, CA, USA. Since the introduction of Invisalign® into the field of orthodontics, research and development of the tools used in transparent aligner therapy have continued, providing increasingly better materials and equipment for its further development (5). One of the most widely used plastic materials with extensive information about its properties is Invisalign SmartTrack™, a material created with clinically proven results exclusively for use in orthodontic aligners. SmartTrack™ aligners are available in treatments performed with Invisalign® (5). It is specifically designed for use as transparent aligners, rather than generic plastic that could be useful in any type of product. This plastic material is made from medical-grade, multi-layer polyurethane resin, free of bisphenol-A (also known as BPA), as well as latex, gluten, and BPS. It is specifically designed to provide more effective tooth movement, clinically proven to move teeth 50% faster and achieve those movements with 75% more predictability. Beyond the material used to make the aligners, it is important to understand the concept of occlusal stability and recognize its absence (5). In an orthodontic treatment, specifically with clear aligners, gradual alignment towards a properly programmed alignment will facilitate better occlusion structure and stability, and consequently, there will be less muscle tension (6). However, can improperly programmed clear aligners be capable of doing the opposite and worsening temporomandibular joint (TMJ) pain and mandibular discomfort? Are aligners sufficient to reduce jaw discomfort? Can aligners cause TMD? are frequent questions that have arisen in this field of dentistry (6). Clear aligner therapy (CAT) has experienced rapid growth and advances in recent years, making it a popular treatment modality in contemporary orthodontics. The muscles of mastication are capable of adapting to the various functional demands imposed on them. These adaptive changes include alterations in their physical size, fiber properties, muscle activity, and contraction force (7). Some studies have shown that patients undergoing CAT have a higher frequency of episodes of teeth clenching when waking up, report sensitivity in the muscles that act on the mandible and produce wear facets on the inner and/or outer surfaces of the aligners. Conversely, orthodontic treatment with fixed appliances may cause patients to avoid tooth contact to reduce dental pain related to orthodontic tooth movement (7). In healthy individuals, there is a correlation between the activity of the sternocleidomastoid muscles and head and neck posture during physiological actions such as swallowing and voluntary maximum clenching. This relationship worsens in patients with temporomandibular disorders (TMD) (8,9). Repetitive clenching of the aligners could be an acquired behavior that acts as a conditioning stimulus to reduce the perception of orthodontic nociceptive stimuli. In fact, the act of clenching the aligners might induce temporary tooth displacement and promote blood flow through the compressed areas of the periodontal ligament, preventing the accumulation of pro - algesic mediators in the periodontal ligament space and promoting pain relief (7). In “The Glossary of Prosthodontic Terms,” bruxism is defined as a parafunctional habit of grinding teeth and as an oral habit that involves grinding or clenching teeth involuntarily, rhythmically or spasmodically, in non-functional movements distinct from mandibular chewing that can lead to occlusal trauma (10). The purpose of this study is to determine whether there is an increase or decrease in dental clenching and/or grinding in patients undergoing treatment with the Invisalign® system. This variable will be assessed through the subjective sensation of overload and muscle fatigue in the masticatory muscles. The justification for the study is aimed at understanding whether this orthodontic technique should be used in patients who have bruxism present prior to orthodontic treatment.

## Material and Methods

This study complies with the Declaration of Helsinki (11) and was approved by the Ethics Committee of the Universidad Europea de Madrid (UEM). It was conducted between September 2021 and May 2022. All participants were patients treated in the orthodontic postgraduate program at the Universidad Europea de Madrid (UEM) and in a private practice in Madrid, Spain. Assuming an α error of 0.05 and a β error of 0.2 in bilateral contrast, 100 patients were needed for the sample to be representative. A total of 100 patients were selected applying the following inclusion criteria: 1) Cooperative patients with correct use of aligners aged between 18 and 45 years; 2) Patients with full permanent dentition except for third molars; 3) Patients with a treatment plan longer than 6 months. The study excluded patients who: 1) Were on treatment with central or peripheral nervous system suppressants or stimulants; 2) Were undergoing treatment with botulinum toxin targeting muscles directly involved in clenching and grinding and patients who underwent orthognathic surgery treatment; 3) Had averaged 5 hours of sleep or less, and 4) Had suffered severe trauma in the orofacial area.

The methodology used was obtained from two sources. First, three questionnaires per patient at three different phases - prior to the start of treatment (T0), at 3 months after the start (T1), and at 6 months after the start of treatment (T2). Second, three clinical examinations (T0, T1, T2) of the masseter, temporal, internal pterygoid, and external pterygoid muscles, assessing the presence or absence of pain upon palpation. The same questionnaire was used at T0, T1, and T2 ( Table 1) and was validated through a pilot study involving 15 professors from the master’s degree in Orthodontics. The reliability of the questionnaire was assessed using Cronbach’s alpha (α = 0.96). The intra-observer error was calculated using Cohen’s Kappa agreement index (k = 0.89). The individual item analysis to observe the presence of reverse items is shown in  Table 2. There are no reverse items. The relationship between dependent and independent variables was analyzed using contingency Tables and the chi-square test. The three questionnaires were analyzed using the Friedman test for repeated measures.

## Results

Data were collected from 100 patients: 58 females (58%) and 42 males (42%). There were 13 patients aged between 18 and 27 years (13%), 62 patients between 28 and 36 years (62%), and 25 patients between 37 and 45 years (27%). Before treatment with aligners, 64% of the patients had bruxism; 26 males (61%) had bruxism and 38 females (65.5%), with no statistically significant differences (*p*>.05). A significant association was found between the presence of bruxism and the age of the patients, being more frequent in those aged between 28 and 36 years (*p*<.05) ( Table 3).

Regarding the use of occlusal splints before starting treatment with aligners, 63% of patients did not use a splint compared to 37% who did. Concerning the item assessing the “feeling of clenching and/or grinding,” there was a general decrease in the sensation of clenching and/or grinding in most patients during the treatment follow-up. This suggests that using Invisalign® aligners might help reduce the feeling of tension or pressure in the oral area while wearing aligners. However, individual variations were observed, as some patients experienced a temporary increase in the sensation of clenching and/or grinding during the first few months of treatment. When comparing T0, T1, and T2, we observed the following changes in the frequency of clenching and/or grinding:

• The frequency of experiencing it 3 to 4 times a week (regularly) decreased by 25%.

• The frequency of experiencing it 1 to 2 times a week (occasionally) increased by 3%.

• The frequency of experiencing it infrequently increased by 20%.

**Figure 1:**
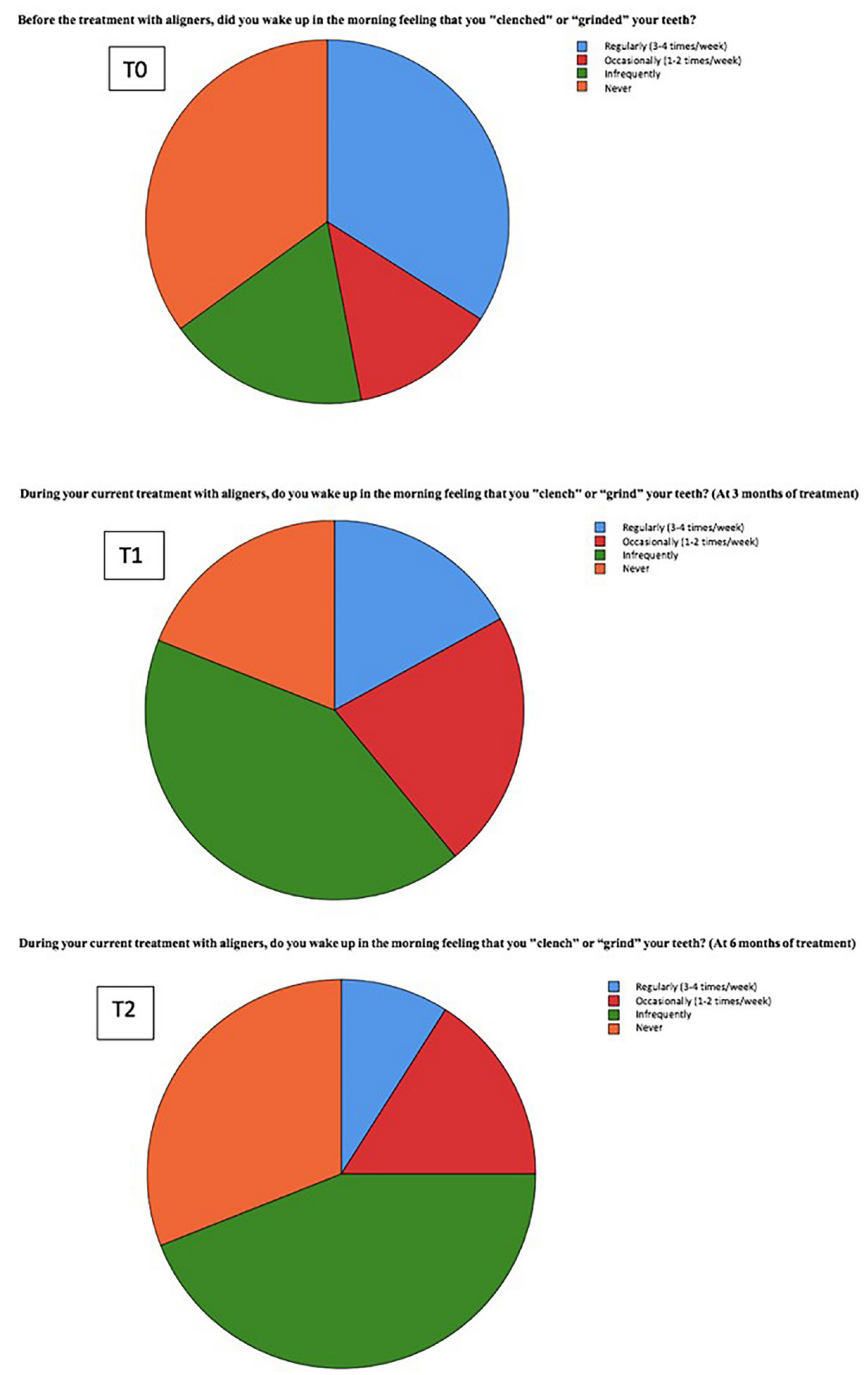
Pie chart of the frequency of the sensation of clenching and/or grinding.

The 25% of patients who reported a regular sensation of teeth clenching and/ or grinding moved to a less frequent sensation, “occasionally, 1 to 2 times a week” and even to “infrequently”. Those who never reported this feeling of bruxism decreased by 4%, which indicates that from the beginning to six months, 4% of patients developed a sensation of clenching and/or grinding (Fig. 1,  Table 4). These results are statistically significant according to the Friedman test.

**Figure 2:**
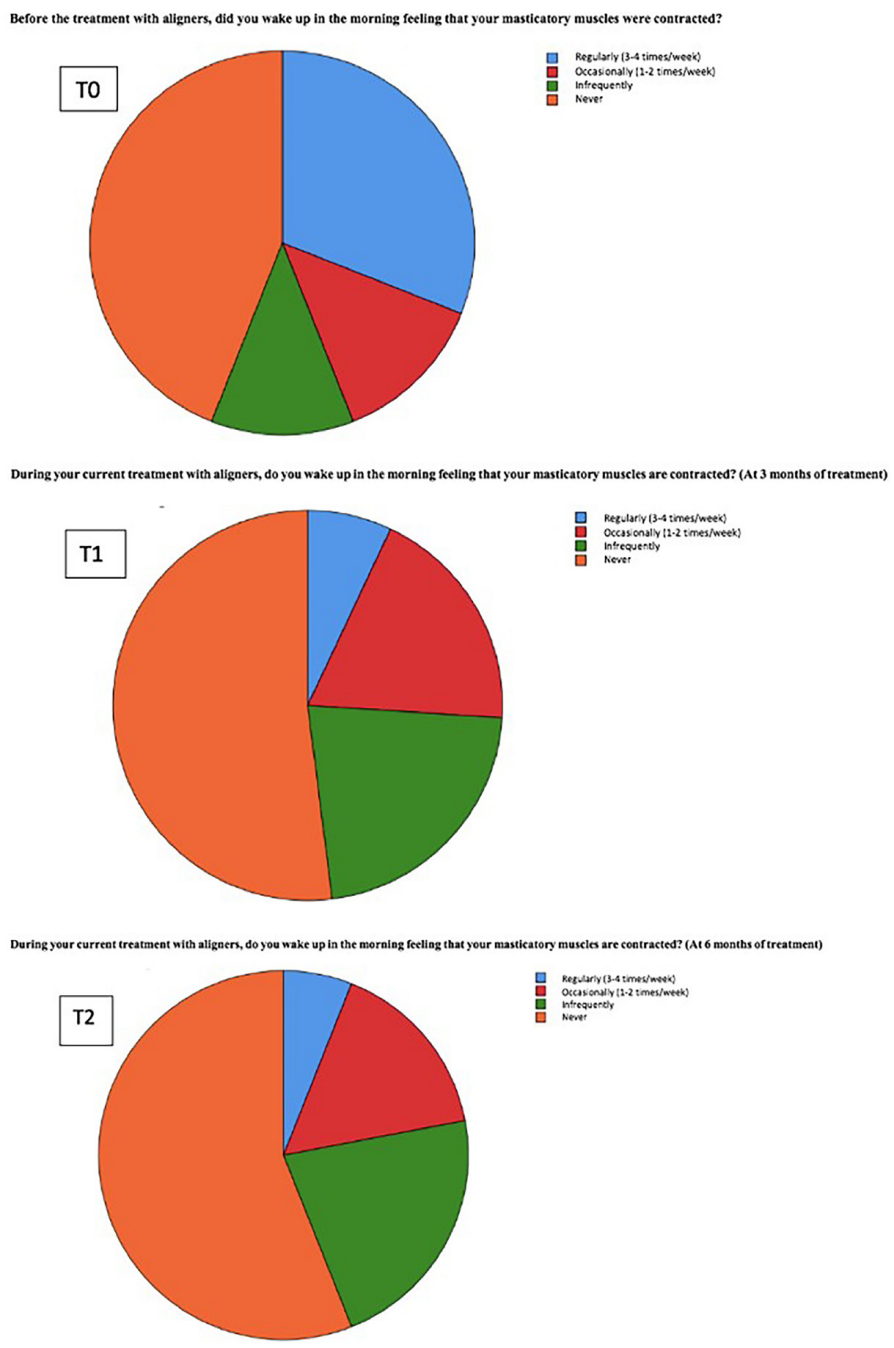
Pie chart of the frequency of the sensation of muscle contraction.

The item “feeling of muscle contraction,” similar to the sensation of clenching and/or grinding, showed a general reduction after treatment with Invisalign® aligners. This suggests a possible muscle relaxation in the oral area while in the treatment with aligners, which may be related to the movement of teeth leading to a correction of dental alignment. Although most patients reported a decrease in this sensation, some patients experienced a temporary increase at the beginning of the treatment (Fig. 2).

In  Table 4, we observe the frequency of the sensation of muscle contraction at T0, T1, and T2. We can see that the frequency of “regularly” (3 to 4 times a week) decreases by 25%, the frequency of “occasionally” (1 to 2 times a week) increases by 3%, and the frequency of “infrequently” rises by 19%. 25% of patients who regularly felt their masticatory muscles contracted moved to experiencing this symptom less frequently (1 to 2 times a week), with the majority transitioning to only experiencing it “infrequently”. The number of patients who never felt muscle contraction increased by 3%, indicating that from the beginning to six months, there has been a 3% decrease in those who feel muscle contraction. These changes are statistically significant ( Table 4).

Regarding the item “pain in the masticatory muscles”, comparing T0, T1, and T2, we observe that the frequency of “regularly” (3/4 times a week) declines by 25%, the frequency of “occasionally” (1 to 2 times a week) increases by 3%, and the frequency of “infrequently” increases by 10%. 25% of patients who regularly felt pain in the masticatory muscles moved to experiencing this symptom less frequently (1 to 2 times a week) and the majority transitioned to the group that no longer felt pain (“never”). The percentage of patients who never experienced pain increased by 11%, meaning they have ceased to experience this pain ( Table 4).

Comparing T0, T1, and T2 in terms of temporomandibular joint (TMJ) pain, we see that the frequency of “regularly” (3/4 times a week) decreases from 22% to 0%, the frequency of “occasionally” (1 to 2 times a week) declines by 3%, and the frequency of “infrequently” rises by 13%. The 22% of patients who regularly experienced TMJ pain moved to experiencing this symptom “occasionally”. The percentage of patients who no longer experienced TMJ pain increased by 12%, meaning that 12% of those with TMJ pain have ceased to experience it ( Table 4).

There are statistically significant differences at 3 and 6 months of wearing aligners regarding teeth clenching and/or grinding while wearing the aligners. After 3 months, 33% of patients reported clenching and/or grinding more while wearing aligners, and after 6 months, this percentage decreased to 19%. After 6 months of treatment with aligners, out of 100 patients, 19 reported more clenching and/or grinding.

Although no statistically significant differences were found, there was a trend toward a reduction in headaches after treatment. This suggests a potential benefit of Invisalign® aligner treatment in reducing headaches associated with temporomandibular joint problems and muscle tension.

Some patients reported changes in facial aesthetics, particularly in the cheek area (masseters), after using Invisalign® aligners. However, these changes were not uniform among patients; some experienced a reduction in cheek thickness, while others experienced an increase.

Regarding the resting lip position, no significant differences were found between 3 and 6 months of treatment. However, significant differences were observed compared to the state before treatment, suggesting that Invisalign® aligners may have a long-term impact on the resting position of the lips. Yet, among these differences, neither the decrease nor the increase in the thickness of the masseter area was predominant.

## Discussion

The results indicated that no significant association was found between bruxism and the gender of the studied patients. It is important to note that, although some previous studies have reported significant differences in the prevalence of bruxism between genders, these results can vary depending on the studied population and other contextual factors. For example, the study conducted by Martynowicz *et al*. (12) showed a higher incidence in males. However, another study carried out by Giulia Savarese *et al*. did not find a significantly different prevalence between genders in their sample of patients (13). In this study, it was observed that the incidence of bruxism appears to decrease with age, suggesting that bruxism may be more prevalent in younger adults than in older individuals. It was found that patients aged 28 to 36 years were most affected by bruxism. This trend is consistent with the conclusions of the American Academy of Sleep Medicine, which also suggest a decrease in the prevalence of bruxism with aging (12). Regarding the frequency of bruxism, the current study revealed that a significant percentage of patients (64%) confirmed having been previously diagnosed with this condition. However, it is important to note that specific data on the incidence of bruxism in the general population were not found. These findings are in line with previous studies that have reported different prevalence rates of bruxism in diverse populations. For example, the study conducted by Savarese *et al*. found that approximately 40.8% of patients considered themselves to have daytime bruxism (13). However, it is important to consider that the perception of bruxism may vary among individuals and that some cases may not have been previously diagnosed.

One of the areas of interest in the study was the sensation of clenching and/or grinding in patients with bruxism. It was observed that the sensation of clenching and/or grinding with aligners compared to not wearing aligners decreased significantly at 3 months and to a greater extent at 6 months of treatment, suggesting an improvement in comfort and muscular relaxation in patients. This finding is consistent with existing literature, which has shown that these oral devices can help reduce muscle activity associated with bruxism and improve related symptoms.

An important feature to highlight is that some patients who had never experienced this sensation of clenching and/or grinding before starting aligner treatment began to feel it after the treatment started, during the first few months, although to a lesser extent, without becoming a habit at 3 months of treatment. This could indicate a sensitization of patients to changes in their occlusion and increased awareness of their muscular activity, which generally decreased at 3 months and more consistently at 6 months of aligner treatment. According to the classic theory of Moller (14), this short-term sensitization effect of oral devices could be explained by a slight change in the position of the jaw, thereby generating an altered modulation of the masseter muscle (15). Occlusal stability appears to be associated with neuromuscular function (16), and the presence of the appliance may have caused an early and temporary effect on the proprioceptive information received by the central nervous system (CNS) (17). A study by Al-Quran FA and Lyons MF shared the idea that this clenching sensation depends on the material the aligner is made of, dividing materials into hard and soft. Hard resin appliances seem to be associated with a decrease in EMG activity of the jaw muscles during sleep when it occurs, whereas soft appliances seem to have the opposite effect (18). From a technical point of view, Giuseppe Siciliani *et al*.’s study indicated that aligners cannot be compared with any devices studied so far. They are neither hard nor soft as they are made of a thin, hard thermoplastic resin sheet (19).

Similar to the results obtained the sensation of clenching and/or grinding, the sensation of muscle contraction also showed a noTable decrease throughout the aligner treatment, with initial bursts of increase which decreased by 3 months and further reduced below initial values at 6 months. In Giuseppe Siciliani *et al*.’s study, short-term changes in muscle activity were evident between T0 (before treatment) and T1 (approximately 1 month after treatment), but returned to baseline or even lower at T2 (approximately 3 months after treatment) (19). This suggests an improvement in muscle relaxation and reduction in tension in patients with bruxism. These findings are consistent with previous studies demonstrating that aligners can help reduce muscle activity and improve comfort in patients with bruxism while in treatment.

Regarding pain in the masticatory muscles, the incorporation of aligner treatment was successful as a continuous decrease was observed. This improvement in masticatory muscle pain has been observed in several studies of patients undergoing orthodontic treatment (20,21). However, the signs and symptoms of masticatory muscle pain are not entirely resolved (22). In the present study, a continuous decrease in masticatory muscle pain was observed at 3 and 6 months of treatment. Some patients who experienced pain regularly saw a reduction in frequency, while others experienced complete disappearance of pain. These findings suggest that the oral devices can effectively alleviate pain associated with bruxism during aligner treatment.

Significant reduction in TMJ pain was observed at 3 and 6 months of treatment. Some patients who experienced pain regularly also saw a reduction in frequency, while others experienced complete disappearance of pain. These findings suggest that treatment with oral devices can effectively reduce pain and improve TMJ function in patients with bruxism. This could be explained by their ability to provide greater or lesser release of clenching and/or grinding, potentially relieving tension in the joint area.

Although there was a trend towards a decrease in headaches, statistically significant results were not found in the current study. Specific data on the relationship between aligner use and headache incidence were not identified. Further research is needed in this area to determine if aligner treatment may impact headaches in patients with bruxism.

Facial aesthetic characteristics and lip position were included in the study due to patient interest while undergoing aligner treatment. Significant differences in facial aesthetics and lip position were found after 3 months of treatment. Some patients noticed changes in cheek thickness, while others noted a different lip position. It is important to note that these aesthetic changes may vary among patients and can be influenced by various factors, including individual facial anatomy and tissue response to occlusal changes.

In summary, the study provided a detailed insight into various aspects of the relationship between bruxism and aligner use. Significant improvements were observed in clenching and/or grinding sensation, masticatory muscles contraction sensation, masticatory muscles pain, and TMJ pain in treated patients. However, further research is needed to understand the relationship with other aspects, such as headaches.

## Conclusions

The treatment with Invisalign® aligners proved effective in reducing bruxism (clenching and/or grinding), muscle contraction and pain in the masticatory muscles, as well as TMJ pain in the majority of patients. Additionally, some beneficial effects were observed in terms of headaches and facial aesthetics. However, further studies are needed to fully understand the long-term effects of Invisalign® aligner treatment on these aspects.

## Figures and Tables

**Table 1:** Questionnaire Reliability.

Frequentist Scale Reliability Statistics	
Estimate	Cronbach's α
Point estimate	0.896
95% CI lower bound	0.863
95% CI upper bound	0.923

**Table 2:** Frequentist Individual Item Reliability Statistics.

Frequentist Individual Item Reliability Statistics	
Items	Item-rest correlation
Feeling of clenching and/or grinding before treatment	0.586
Feeling of muscle contraction before treatment	0.613
Pain in masticatory muscles before treatment	0.607
Pain in the TMJ (temporomandibular joint) before treatment	0.591
Feeling of clenching and/or grinding 3 months after starting treatment	0.672
Feeling of muscle contraction 3 months after starting treatment	0.734
Pain in the masticatory muscles 3 months after starting treatment	0.816
Pain in the TMJ 3 months after starting treatment	0.793
After 3 months of treatment, do you find that you clench or grind your teeth more when wearing your aligners?	0.299
After 3 months of treatment, have your headaches increased?	0,504
After 3 months of treatment, have you noticed aesthetic differences?	0.397
After 3 months of treatment, have you noticed a difference in the resting position of the lips?	0.088
Feeling of clenching and/or grinding 6 months after starting treatment	0.742
Feeling of muscle contraction 6 months after starting treatment	0.706
Pain in masticatory muscles 6 months after starting treatment	0.775
Pain in the TMJ 6 months after starting treatment	0.787
After 6 months of treatment, do you feel you clench or grind your teeth more when wearing your aligners?	0.210
After 6 months of treatment, have your headaches increased?	0.404
After 6 months of treatment, have you noticed aesthetic differences?	0.305
After 6 months of treatment, have you noticed a difference in the resting position of the lips?	0.081

**Table 3:** Association of sociodemographic data with bruxism.

	N=64	Percentage	Test contrast
			p-value
SEX/BRUXISM			.833 (ns)
Female	26	66%	
Male	38	61%	
AGE/BRUXISM			.000**
18-27	13	100%	
28-36	26	41.9%	
37-45	25	100%	

(ns) = not significant; (**): very significant

**Table 4:** Descriptive statistics and chi-test.

N=100	T0	T1	T2	χ²
	Frequency (%)	Frequency(%)	Frequency (%)	P value
"Clenching/Grinding Teeth" feeling:				
Regularly (3-4 times/week)	34(34%)	17(17%)	9(9%)	.0001
Occasionally (1-2 times/week)	13(13%)	22(22%)	16(16%)	
Infrequently	18(18%)	42(42%)	44(44%)	
Never	35(35%)	19(19%)	31(31%)	
Feeling of jaw muscle contraction:				
Regularly (3-4 times/week)	34(34%)	10(10%)	9(9%)	.0001
Occasionally (1-2 times/week)	13(13%)	26(26%)	16(16%)	
Infrequently	15(15%)	26(26%)	34(34%)	
Never	38(38%)	38(38%)	41(41%)	
Jaw muscle pain/ Pain in the masticatory muscles:				
Regularly (3-4 times/week)	31(31%)	7(7%)	6(6%)	.0001
Occasionally (1-2 times/week)	13(13%)	19(19%)	16(16%)	
Infrequently	12(12%)	22(22%)	22(22%)	
Never	44(44%)	52(52%)	56(56%)	
Pain in the TMJ:				
Regularly (3-4 times/week)	22(22%)	4(4%)	0(0%)	.0001
Occasionally (1-2 times/week)	15(15%)	12(12%)	12(12%)	
Infrequently	16(16%)	32(32%)	29(29%)	
Never	47(47%)	52(52%)	59(59%)	
Do you feel you clench or brux more with aligners?:				
Yes		33(33%)	19(19%)	.0001
I don't know		3(3%)	10(10%)	
No		64(64%)	71(71%)	
Headache with aligners?				
Yes, more than 15 days per month		0(0%)	0(0%)	.206
Yes, less than 15 days per month		0(0%)	3(3%)	
Yes, 1-2 times per week		19(19%)	9(9%)	
No, I do not have headaches		81(81%)	88(88%)	
Aesthetic differences with aligners?				
Yes, I have noticed a decrease in volume behind the cheeks.		3(3%)	9(9%)	.003
Yes, I have noticed an increase in volume behind the cheeks.		7(7%)	9(9%)	
No, I have not noticed any differences.		90(90%)	82(82%)	
Have you noticed a difference in the resting position of your lips?				
Yes, I have noticed that my lips are slightly opened at rest.		27(27%)	27(27%)	.083
Yes, I have noticed that my lips are more closed at rest.		12(12%)	9(9%)	
No, I have not noticed any differences in the resting position of my lips.		61(61%)	64(64%)	

## Data Availability

The datasets used and/or analyzed during the current study are available from the corresponding author.
